# Predictors of Smoke-Free Policies in Affordable Multiunit Housing, North Carolina, 2013

**DOI:** 10.5888/pcd12.140506

**Published:** 2015-05-14

**Authors:** Anna Stein, Janet Suttie, Laura Baker, Robert Agans, Wei Xue, J. Michael Bowling

**Affiliations:** Author Affiliations: Anna Stein, Janet Suttie, Laura Baker, North Carolina Division of Public Health, Raleigh, North Carolina; Robert Agans, Wei Xue, J. Michael Bowling, University of North Carolina at Chapel Hill, Chapel Hill, North Carolina.

## Abstract

**Introduction:**

Smoke-free policies can effectively protect nonsmokers from secondhand smoke (SHS) exposure in multiunit housing. We surveyed all affordable multiunit housing properties in North Carolina to determine the statewide prevalence of smoke-free policies and to identify predictors of smoke-free policies.

**Methods:**

Representatives of affordable housing properties in North Carolina completed a mailed or online survey during June through October 2013. The primary outcome measure was presence of a smoke-free policy, defined as prohibiting smoking in all residential units. We used χ^2^ analysis and multivariate logistic regression to identify correlates of smoke-free policies.

**Results:**

Of 1,865 eligible properties, responses were received for 1,063 (57%). A total of 16.5% of properties had policies that prohibited smoking in all residential units, while 69.6% prohibited smoking in indoor common areas. In multivariate analysis, an increase in the number of children per unit was associated with a decrease in the odds of having a smoke-free policy at most properties. Newer properties across all company sizes were more likely to have smoke-free policies. Accessing units from interior hallways predicted smoke-free policies among medium-sized companies.

**Conclusion:**

More smoke-free policies in affordable multiunit housing are needed to protect vulnerable populations, particularly children, from SHS exposure. Public health professionals should continue to educate housing operators about SHS and the benefits of smoke-free policies at all properties, including older ones and ones where units are accessed from outside rather than from an interior hallway.

## Introduction

The home is a primary source of secondhand smoke (SHS) exposure (1). Home SHS exposure is of particular concern in multiunit housing (MUH), where SHS can easily travel between units (1,2). Almost half of MUH residents with personal smoke-free home rules still experience SHS infiltration in their homes (3). Residents of affordable housing are at particular risk because of higher rates of smoking among low-income populations (4) and decreased ability to move if affected by SHS (5). Many residents in affordable housing are children, elderly, or disabled, and they may experience more detrimental health effects because of SHS exposure (1).

Smoke-free policies can protect nonsmokers from SHS exposure in MUH (6,7), but surveys of property managers in several localities have found that few of their properties have 100% smoke-free residential units, with a range of 9% to 16% (8–10). As public health professionals promote smoke-free housing, additional studies are needed to monitor the availability of smoke-free housing over time. Most studies of smoke-free policies in MUH have assessed attitudes of individual housing operators, who may be affiliated with multiple properties, rather than sampling at the property level to determine policy status (8,11–13). The few studies at the property level had small sample sizes or low response rates (9,10). The purpose of this study was to determine the prevalence of smoke-free policies among affordable MUH properties in North Carolina and to identify factors associated with smoke-free policies. This research project was supported by the Centers for Disease Control and Prevention (CDC) through the Community Transformation Grant.

## Methods

This study was a cross-sectional, observational study. Our sample of affordable housing properties included all multiunit properties in North Carolina that receive site-based federal subsidies or state low-income housing tax credits. This sample comprised properties owned by for-profit and nonprofit private owners and did not include public housing properties. We defined MUH as housing with more than 1 living unit and excluded properties classified as group homes.

This study was an establishment survey, with the unit of analysis being the property itself and not the individual answering the survey. In some cases, a single respondent completed multiple surveys because he or she managed more than 1 property; however, all survey questions were objective questions about policies and events occurring at the property and none assessed respondent attitudes.

We created a sampling frame of affordable housing properties in North Carolina by obtaining lists from the US Department of Agriculture’s (USDA’s) Rural Development program, the US Department of Housing and Urban Development (HUD), and the North Carolina Housing Finance Agency (NCHFA), the state’s purveyor of low-income housing tax credits. We cross-checked this frame with lists of properties from 8 large affordable housing management companies and added missing properties. After removing duplicates, we identified 1,865 properties, all of which were eligible to participate in this census survey.

We developed the survey instrument by reviewing previous MUH operator surveys and obtaining input from smoke-free MUH researchers. We also consulted affordable housing property managers, who provided feedback regarding use of industry-specific terms and appropriateness of survey response categories. We pretested the survey with 18 property managers and made revisions to eliminate confusing wording and skip patterns and reduce respondents’ difficulty with finding information needed to answer the questions.

### Data collection

The Office of Human Research Ethics at the University of North Carolina at Chapel Hill (UNC-CH) ruled that this study did not constitute human subjects research under federal regulations and did not require its approval. We called properties to confirm the appropriate respondents’ names and contact information and their preference for an online or paper survey. For large companies, we contacted corporate headquarters to determine the most appropriate survey respondent for their properties.

The Carolina Survey Research Laboratory (CSRL) at UNC-CH collected data from June to October 2013. Respondents who chose to complete the survey online received a mailed letter containing the URL and password for the survey. Respondents who preferred paper surveys were mailed a questionnaire in booklet form with a prepaid return envelope. The CSRL contacted nonrespondents by telephone during July and August 2013 and sent them either online survey information or mailed questionnaires by August 30, 2013. Several medium and large companies (defined below) instructed their property managers to participate in the survey and contacted nonrespondents twice during the data collection period to encourage participation. Respondents who chose to be compensated received a check for $25.

### Data analysis

To ensure representation among small companies and single owners, we stratified and weighted properties by management company size. Using tertiary cut points, we defined as small those companies managing 1 to 27 properties in North Carolina, as medium those managing 28 to 79, and as large those managing 80 to 171. We produced weights as the inverse stratum-specific sampling rates and then adjusted for differential response based on stratum-specific response rates. SAS software (version 9.2) (SAS Institute, Inc) was used in all statistical analyses and reported significance at the .05 level.

We used χ^2^ goodness-of-fit tabulations to compare characteristics such as year built and location, subsidy type and source, and type of access to units in smoke-free and smoking-allowed properties. We used logistic regression to identify relationships between a smoke-free policy and possible predictor variables. Variables included in the logistic regression model were those that were significant in bivariate analysis or identified by North Carolina housing operators as being important in determining whether to adopt smoke-free policies. The model included the following predictor variables: year built (in or before 2001, or after 2001); company size; type of access to residential units (from an interior hallway, from outside, or a combination); number of stories in residential buildings (1 or 2 stories or ≥3 stories); and number of children per unit (number of children per property divided by the number of residential units per property). The quadratic term for number of children per unit was also included because it indicated curvilinearity in the relationship between the number of children per unit and the log odds of a property’s being smoke-free. Family subsidy status was included in an early version of the model but was not significant and not included in the final model.

## Results

A total of 1,063 completed surveys were received for the 1,865 eligible properties, for a response rate of 57%. Almost two-thirds (62.3%) of the respondents completed the survey online. Most surveys (87%) were completed by on-site managers.

The responding properties had an average of 55.2 units and 8.0 buildings per property (weighted numbers) (Table 1). Three-quarters of properties were built before 2002. Most properties received subsidies designated for elderly households (58.6% weighted percentage) or disabled households (66.5% weighted percentage), and a minority (41.9% weighted percentage) received subsidies designated for families (a catch-all category with income requirements, not familial status requirements) (15,16). Properties were subsidized by NCHFA tax credits (45.8% unweighted percentage), HUD (38.4% unweighted percentage), and USDA (36.6% unweighted percentage). The greatest percentage of responding properties were managed by large companies (36.7% unweighted percentage), followed by small (33.8% unweighted percentage) and medium (29.5% unweighted percentage) companies.

**Table 1:** Characteristics of Multiunit Affordable Housing Properties, North Carolina,^a^ 2013

Characteristic	Unweighted	Weighted^b^
**Property size, mean (95% CI)**		
No. of units (n = 1,063)	51.2	55.2 (51.7–58.6)
No. of residential buildings (n = 1,061)	7.9	8.0 (7.0–8.9)
**Year property was built (n = 1,035), % (95% CI)**		
≤2001	74.7	75.0 (72.2–77.8)
>2001	25.3	25.0 (22.2–27.8)
**Subsidy type, % (95% CI)**		
Family subsidies (n = 1,051)	41.9	40.2 (37.1–43.3)
Elderly subsidies (n = 1,054)	58.6	57.3 (54.2–60.5)
Disability subsidies (n = 1,055)	66.5	64.6 (61.5–67.6)
**Subsidy source, % (95% CI)**		
USDA Rural Development	36.6	31.4 (28.8–33.9)
HUD	38.4	43.7 (40.7–46.6)
NCHFA tax credits	45.8	43.2 (40.1–46.2)
**Management company size^c^ (n = 1, 063), % (95% CI)**		
Small	33.8	47.1 (43.8–50.3)
Medium	29.5	29.8 (26.9–32.6)
Large	36.7	23.2 (20.9–25.4)
**MSA status^d^ of county in which property is located (n = 1,063), % (95% CI)**		
MSA	62.8	64.9 (62.0–67.9)
Non-MSA	37.2	35.1 (32.1–38.0)

Abbreviations: CI, confidence interval; HUD, US Department of Housing and Urban Development; MSA, metropolitan statistical area; NCHFA, North Carolina Housing Finance Agency; USDA, US Department of Agriculture.

a All multiunit properties in North Carolina that receive site-based federal subsidies or state low-income housing tax credits. This sample comprised properties owned by for-profit and nonprofit private owners and did not include public housing properties.

b Because of rounding of weighted percentages, some totals may not sum to 100%.

c Small companies were defined as managing 1–27 properties, medium companies were defined as managing 28–79 properties, and large companies were defined as managing 80–171 properties.

d Office of Management and Budget (14).

Bivariate analysis indicated a relationship between subsidy source and year built. HUD properties were older than NCHFA properties (*P* < .001), with 48.5% (95% CI, 44.9%–52.0%) of properties built before 2002 receiving HUD subsidies and 34.2% (95% CI, 30.7%–37.7%) receiving NCHFA tax credits.

### Prevalence of smoke-free policies

Most properties (81.2%; 95% CI, 78.8%–83.5%) allowed smoking in all residential units, while 16.5% (95% CI, 14.2%–18.7%) did not allow smoking in any residential units, and 2.4% (95% CI, 1.4%–3.3%) allowed smoking in some residential units. Of the very small percentage of properties that allowed smoking in some residential units, 73.6% (95% CI, 62.7%–84.6%) allowed current residents to smoke under a “grandfather” policy, with the intention of being completely smoke-free once all grandfathered smokers had moved out. Among properties prohibiting smoking in residential units, 58.3% (95% CI, 52.4%–64.3%) had converted to smoke-free and 41.7% (95% CI, 35.7%–47.6%) had opened as smoke-free. Most properties banned smoking in indoor common areas (69.6%; 95% CI, 66.7%–72.6%), while few properties prohibited smoking in outdoor common areas (10.8%; 95% CI, 8.9%–12.6%) or outdoor private areas (7.9%; 95% CI, 6.3%–9.5%).

### Characteristics of smoke-free properties

For purposes of comparing smoke-free and smoking-allowed properties, we categorized properties as having a smoke-free policy if smoking was not allowed in any residential units, and smoking-allowed if smoking was allowed in some or all residential units. Children were less likely than adults to live in properties with smoke-free policies, with 10.5% (95% CI, 5.6%–15.4%) of children living in smoke-free properties and 15.1% (95% CI, 10.6%–19.6%) of adults doing so.

In bivariate analysis, the following variables were significantly associated with smoke-free policy status (Table 2): year built, subsidy type, subsidy source, company size, and type of access to residential units. A greater proportion of properties built after 2001 had smoke-free policies compared with older properties (*P* = .01). Properties without family subsidies were more likely to be smoke-free than those with family subsidies (*P* = .01). There was no association between smoke-free policies and receiving USDA subsidies, while HUD subsidies were negatively associated with smoke-free policies (*P* < .001) and NCHFA tax credits were positively associated with smoke-free policies (*P* < .001). More medium-sized companies had smoke-free policies than large or small companies (*P* = .01). Type of access to residential units was also associated with having a smoke-free policy (*P* < .001); a larger proportion of properties with units accessed from the interior had smoke-free policies (23%) than properties with units accessed from outdoors (13%). Number of stories in residential buildings, metropolitan statistical area status of the county in which the property is located (14), and receiving subsidies designated for the elderly or disabled were not associated with smoke-free policy status.

**Table 2:** Characteristics of Smoking-Allowed and Smoke-Free^a^ Affordable Multiunit Housing Properties, North Carolina,^b^ 2013

Characteristic	Smoking-Allowed (n = 886), Weighted % (95% CI)	Smoke-Free (n = 177), Weighted % (95% CI)	*P* Value^c^
**Total**	83.5 (81.3–85.8)	16.5 (14.2–18.7)	NA
**Year built**			
<1981	89.3 (85.3–93.3)	10.7 (6.7–14.7)	.01
1981–1989	89.8 (86.0–93.6)	10.2 (6.4–14.0)	
1990–2001	86.8 (82.5–91.1)	13.2 (8.9–17.5)	
>2001	70.3 (64.6–76.0)	29.7 (24.0–35.4)	
**USDA Rural Development subsidy**			
Yes	85.0 (81.4–88.7)	15.0 (11.3–18.6)	.36
No	82.8 (80.0–85.7)	17.2 (14.3–20.0)	
**HUD subsidy**			
Yes	89.5 (86.6–92.5)	10.5 (7.5–13.4)	<.001
No	78.9 (75.6–82.1)	21.1 (17.9–24.4)	
**NCHFA tax credits**			
Yes	78.8 (75.0–82.6)	21.2 (17.4–25.0)	<.001
No	87.1 (84.4–89.9)	12.9 (10.1–15.6)	
**Family subsidy**			
Yes	88.1 (84.9–91.3)	11.9 (8.7–15.1)	.01
No	80.3 (77.1–83.5)	19.7 (16.5–22.9)	
**Elderly subsidy**			
Yes	85.1 (82.3–87.9)	14.9 (12.1–17.7)	.11
No	81.3 (77.5–85.1)	18.7 (14.9–22.5)	
**Disability subsidy**			
Yes	82.5 (79.6–85.4)	17.5 (14.6–20.4)	.24
No	85.4 (81.7–89.2)	14.6 (10.8–18.3)	
**Company size^d^**			
Small	85.4 (81.8–89.0)	14.6 (11.0–18.2)	.01
Medium	77.9 (73.7–82.1)	22.1 (17.9–26.3)	
Large	86.9 (83.6–90.2)	13.1 (9.8–16.4)	
**Type of access to residential units**			
Interior hallway	77.0 (71.6–82.4)	23.0 (17.6–28.4)	<.001
Outside	86.7 (84.2–89.3)	13.3 (10.7–15.8)	
Both	74.9 (65.4–84.4)	25.1 (15.6–34.6)	
**Residential buildings with 3 or more stories**			
Yes	82.4 (76.8–88.0)	17.6 (12.0–23.2)	.65
No	83.8 (81.3–86.3)	16.2 (13.7–18.7)	
**MSA status^e^ of county in which property located**			
MSA	84.1 (80.3–88.0)	15.9 (12.0–19.7)	.71
Non-MSA	83.2 (80.4–86.0)	16.8 (14.0–19.6)	

Abbreviations: CI, confidence interval; HUD, US Department of Housing and Urban Development; MSA, metropolitan statistical area; NA, not applicable; NCHFA, North Carolina Housing Finance Agency; USDA, US Department of Agriculture.

a Properties were defined as “smoking-allowed” if they allowed smoking in some or all residential units, and “smoke-free” if smoking was banned in all residential units.

b All multiunit properties in North Carolina that receive site-based federal subsidies or state low-income housing tax credits. This sample comprised properties owned by for-profit and nonprofit private owners and did not include public housing properties.

c χ^2^ test comparing characteristic categories of smoking-allowed and smoke-free properties.

d Small companies were defined as managing 1 to 27 properties, medium companies were defined as managing 28 to 79 properties, and large companies were defined as managing 80 to 171 properties.

e Office of Management and Budget (14).

### Predictors of smoke-free policies

In the multivariate model of predictors of smoke-free policies, examination of 2-way interactions (likelihood ratio test *P* < .05) between company size and other variables (number of children per unit, the quadratic term for number of children per unit, year built, type of access to residential units, and number of stories) indicated evidence of effect moderation. Because of this interaction effect, we have presented the effect of the predictor variables on smoke-free policies by company size (Table 3).

**Table 3:** Predictors of Having a Smoke-Free Policy^a^ Among Multiunit Affordable Housing Properties by Size of Management Company, North Carolina,^b^ 2013

Variables	Small Company^b^		Medium Company^d^		Large Company^e^	
	Odds Ratio (95% CI)	*P* Value^f^	Odds Ratio (95% CI)	*P* Value^f^	Odds Ratio (95% CI)	*P* Value^f^
**Children**						
Number of children per unit^g^	0.7 (0.2–2.1)	.50	0.1 (0.0–0.6)	.02	0.1 (0.0–0.4)	.003
Quadratic term for the number of children per unit	1.1 (0.7–1.7)	.67	3.9 (0.9–15.2)	.05	2.7 (1.2–6.0)	.02
**Year built**						
≤2001	0.3 (0.1–0.5)	<.001	0.4 (0.2–0.8)	.005	0.2 (0.1–0.3)	<.001
>2001	1 [Reference]					
**Access to residential units**						
Interior hallway	0.6 (0.3–1.3)	.22	3.4 (1.7–7.0)	<.001	1.1 (0.4–3.0)	.85
Outside or both	1 [Reference]					
**Number of stories in residential buildings**						
1 or 2	1.2 (0.5–2.5)	.70	3.7 (1.4–10.1)	.01	0.3 (0.1–0.7)	.006
3 or more	1 [Reference]					

Abbreviation: CI, confidence interval.

a Properties were defined as having a smoke-free policy if they did not allow smoking in any residential units.

b All multiunit properties in North Carolina that receive site-based federal subsidies or state low-income housing tax credits. This sample comprised properties owned by for-profit and nonprofit private owners and did not include public housing properties.

c Small companies were defined as managing 1 to 27 properties in North Carolina.

d Medium companies were defined as managing 28 to 79 properties in North Carolina.

e Large companies were defined as managing 80 to 171 properties in North Carolina.

f Logistic regression model of predictors of smoke-free policies by management company size.

g Number of children per property divided by the number of units per property.

An increasing number of children per unit was associated with decreasing odds of a smoke-free policy at properties managed by both medium and large companies. The number of children per unit was inversely related to the presence of a smoke-free policy until reaching 1.9 children per unit among properties managed by medium companies and 2.5 children per unit among properties managed by large companies, shifting to a positive relationship at higher numbers. Properties where an increasing number of children per unit was associated with decreasing odds of a smoke-free policy included 76.7% of properties managed by medium companies and 89.8% of properties managed by large companies.

Several other variables predicted smoke-free policy status in multivariate analysis. Properties managed by all company sizes had lower odds of having a smoke-free policy if they were built before 2002. Type of access to residential units predicted smoke-free policy status among properties managed by medium companies: smoke-free policies were more likely among properties with units accessed by interior hallways (odds ratio, 3.4).

## Discussion

This study was the first statewide survey at the property level to assess the availability of smoke-free policies in affordable MUH. While most properties banned smoking in indoor common areas, few prohibited smoking in residential units, suggesting significant potential for exposure to SHS for residents. Although North Carolina is a tobacco-growing state, this prevalence of smoke-free policies is similar to findings from operator surveys in other areas of the country (8–10). Few properties banned smoking in outdoor private areas, raising the concern that even when residents are protected from smoke transfer between units indoors, they may still be exposed to smoke drifting into their units from neighbors’ porches, patios, or balconies.

We found that children in North Carolina affordable housing are at increased risk of SHS exposure. An increasing concentration of children was associated with decreasing odds of having a smoke-free policy at most properties. Also, children were more likely to live in smoking-allowed properties than adults; however, the strength of this latter finding is in question because of overlapping confidence intervals. This study adds to the small body of literature regarding children’s SHS exposure in MUH. Children living in MUH have significantly higher levels of exposure to SHS than children living in detached houses (17). Supportive of our data, the 2011–2012 National Health and Nutrition Examination Survey demonstrated that prevalence of SHS exposure was significantly higher among children, particularly non-Hispanic black children, than among adults (18). In contrast to our results, a 2010 national survey of MUH residents found that respondents with children were more likely to report living in smoke-free buildings; however, this data also had overlapping confidence intervals (19). A 2007–2009 New York study found that MUH residents with children were more likely to report SHS entering their personal living space than those without children (20), while a 2011 national survey found that residents with children were more likely to report smoke incursions in their buildings but less likely to report incursions in their units (21). Several studies of smoke-free home rules have found a greater likelihood of personal home smoking restrictions in households with children (20,22–25); however, regardless of individual home restrictions, children in MUH will continue to be exposed to SHS if they live in buildings where smoking is allowed. Our survey suggests the need to prioritize smoke-free policy messaging and outreach to housing for families with children.

Older properties across all management company sizes were less likely to have smoke-free policies. This finding, also observed in a New York study of MUH (8), suggests there may be fewer barriers to opening properties smoke-free than converting existing properties. Public health professionals could prioritize outreach and technical assistance to older properties, targeting support where it is most needed. More than half of smoke-free properties in this study were converted to smoke-free rather than opening smoke-free, suggesting that conversion of existing properties is feasible.

Our study also found an association between the existence of a smoke-free policy at a property and its source of subsidy: HUD-subsidized properties were less likely to be smoke-free, while properties subsidized by NCHFA tax credits were more likely to be smoke-free. This difference may be attributed to the age of the property: HUD properties were older than properties that received tax credits, which are used to finance new construction or renovations (26). Similar to our results, findings from a South Dakota study showed that HUD properties were less likely to have smoke-free policies (11). HUD is taking proactive efforts to encourage smoke-free policies among its subsidized properties (27) and has published an action guide for smoke-free policy implementation (28), so these numbers may improve in the future.

We examined variables not tested in other studies, including management company size and type of access to residential units. Regarding management company size, small and large companies were less likely to have smoke-free policies than medium companies. Small companies may lack knowledge about trends in smoke-free policies, and large companies may be reluctant to convert a large portfolio of properties to smoke-free. More research is needed to determine reasons for this finding and to guide targeted outreach to promote smoke-free policies among small and large management companies. Regarding type of access to residential units, buildings with units accessed from the outside were less likely to have smoke-free policies than units accessed from an interior hallway; interior hallway access predicted smoke-free policies among medium companies. Housing operators may believe there is less smoke transfer between units that do not share a common indoor hallway, so that properties of this style are a lower priority for smoke-free conversion. North Carolina housing operators suggested adding this question to the survey, demonstrating the importance of engaging stakeholders to identify relevant fields of inquiry. Public health professionals should emphasize in their communications to housing operators that residents of apartments accessed from outdoors would benefit from smoke-free policies as well, because smoke can travel between these units through shared walls and ceilings and inside from outdoor balconies and patios.

Strengths of this study include incorporating input from housing industry professionals into the survey design, sampling at the property level, using government lists to create the sampling frame, and having a large sample size and a high response rate in comparison to previous operator surveys. However, this study has several limitations. First, the study examined only affordable housing properties and the results may not be generalizable to market-rate housing. It is possible that market-rate housing has a higher prevalence of smoke-free policies because of lower smoking rates among higher income individuals (29). This question merits further study. Moreover, our results may not be generalizable to affordable housing properties in other states. However, North Carolina’s smoking rates are near the national average (4,29), and if acceptance of smoke-free policies is tied to smoking prevalence, these smoke-free policy rates may also be indicative of national trends. Another limitation is that this study relied on operator report and did not require separate verification of data. However, we pilot tested the survey with property managers who assured us they knew or could readily access all the information requested.

More smoke-free policies in affordable MUH are needed to protect vulnerable populations, particularly children, from SHS exposure. The availability of smoke-free policies in affordable housing properties in North Carolina is low, though increasing. After data collection was completed, 2 large management companies converted their properties to smoke-free in 2014, increasing the percentage of smoke-free properties in North Carolina from 16.5% to 27.9% in the period of a year. To ensure this trend continues in North Carolina and is replicated throughout the country, public health professionals should continue to educate housing operators about the benefits of smoke-free policies at all multiunit properties.
